# Effects of Preharvest Methyl Jasmonate and Salicylic Acid Treatments on Growth, Quality, Volatile Components, and Antioxidant Systems of Chinese Chives

**DOI:** 10.3389/fpls.2021.767335

**Published:** 2022-01-07

**Authors:** Cheng Wang, Jing Zhang, Jianming Xie, Jihua Yu, Jing Li, Jian Lv, Yanqiang Gao, Tianhang Niu, Bakpa Emily Patience

**Affiliations:** College of Horticulture, Gansu Agricultural University, Lanzhou, China

**Keywords:** methyl jasmonate, salicylic acid, antioxidant activity, quality, volatile compositions

## Abstract

Methyl jasmonate (MeJA) and salicylic acid (SA) regulate the production of biologically active compounds in plants and stimulate the accumulation of plant aromatic substances. However, the underlying mechanisms of how MeJA and SA influence characteristic flavor compounds and the antioxidant activity of vegetables are poorly understood. Five MeJA and SA concentrations were used to investigate the dose-dependent effects of these phytohormones on the dry and fresh weight; chlorophyll abundance; the contents of vitamin C, soluble protein, and sugar, nitrate, total phenols, flavonoids, volatile components, and enzymatically produced pyruvic acid; and antioxidant activity in Chinese chive. We found that MeJA and SA at concentrations of 500 and 150 μM, respectively, significantly increased the levels of total chlorophyll, phenols and flavonoids, vitamin C, and volatile components and significantly reduced the accumulation of nitrate. In addition, compared with the control, 500 μM of MeJA significantly increased the soluble sugar and protein content, and 150 μM SA significantly increased the dry and fresh weight of Chinese chive. Furthermore, these concentrations of MeJA and SA significantly increased the enzymatic pyruvate content and the amount of sulfide and aromatic volatile compounds and improved the characteristic flavor compounds. The 2,2-diphenyl-1-picrylhydrazyl radical scavenging capacity, Trolox-equivalent antioxidant capacity, and ferric-reducing antioxidant capacity were significantly improved after a preharvest treatment with 500 μM MeJA and 150 μM SA, which could improve the antioxidant activity, thus improving the postharvest quality and preservation characteristics of Chinese chives. Taken together, a preharvest treatment with 500 μM MeJA and 150 μM SA is optimal to improve the growth, quality, antioxidant activity, and flavor of Chinese chive, thereby enhancing its commercial value.

## Introduction

Fruits and vegetables contain a large amount of nutrients required by the human body, and their minerals, vitamins, and antioxidants are important for maintaining human health. As such, plants play a key role in maintaining a balanced diet and in well-being (Gbska et al., 2020). Phenols, carotenoids, alkaloids, and organosulfur compounds are the main phytochemical categories (Liu, 2004). Organosulfur compounds are the main flavor precursors of Allium plants, and they endow Allium vegetables with great nutritional and medicinal value (Jones, 2004; Yabuki et al., 2010). Flavonoids have antifungal activity in plants and are therefore involved in pathogen defense (Napal et al., 2009). Phenolics and flavonoids play an important role as antioxidants in raw food materials (Nayak et al., 2013; Ahmed and Eun, 2017). The nutritional value of vegetables is also greatly improved (Soppelsa et al., 2018).

Chinese chive (*Allium tuberosum* Rottler ex Spreng.) is a popular vegetable in China. As a commercial crop, Chinese chives are cultivated all over China (Mau et al., 2001). Similar to that of garlic (*Allium sativum* L.), Chinese chive has a distinctive odor due to volatile compounds and is rich in nutrients, such as vitamins, proteins, carbohydrates, and cellulose (Rose et al., 2005; Gao et al., 2018). Studies have shown that it is also rich in saponins, alkaloids, phenylpropanoid glucoside, polysaccharides, phenols, flavonoids, and sulfur compounds (Zhang et al., 2016; Tang et al., 2017). This is because Chinese chive can produce sulfur-containing metabolites-S-alk(alkene) cysteine sulfoxides (CSOs), which have an extremely high nutritional and medicinal value (Rabinowitch and Currah, 2002). CSOs are the main flavor precursors of Allium vegetables (Liu et al., 2021). The flavor of Allium plants is produced by the hydrolysis of CSOs by alliinase when the cells are ruptured, and the amount of pyruvate produced in the hydrolysis reaction is equivalent to that of CSOs, which can be used to evaluate the flavor of Chinese chive (Wall and Corgan, 1992). Chinese chive is a vegetable that has been used for both food and in medicine (Leelarungrayub et al., 2006; Bernaert et al., 2012). It has anti-inflammatory, anticancer, antibacterial, and antioxidant activities, which are mainly related to its sulfur-containing compounds (Rattanachaikunsopon and Phumkhachorn, 2009; Hong et al., 2014; Zeng et al., 2017). Additionally, Chinese chives are considered as a health food that can improve the kidney function (Masuda and Mihara, 1988; Block et al., 1992), and they are used for sexual enhancement, nocturnal emission, abdominal pain, and diarrhea in traditional Chinese medicine (Jiangsu, 1979). Therefore, increasing the nutritional quality, antioxidant activity, and organosulfur compounds could be a good strategy to enhance the potential health benefits of Allium vegetables, including Chinese chives.

Jasmonic acid (JA) and methyl jasmonate (MeJA) and their derivatives are ubiquitous lipid-based plant hormones (Sheard et al., 2010). MeJA is a natural plant growth regulator, which has a wide range of physiological effects on plant growth, development, and abiotic tolerance, such as postharvest preservation, quality improvement, secondary metabolite synthesis, and stress response (Meir et al., 1996; Delker et al., 2006; Donati et al., 2018). Based on these reports, MeJA treatment can regulate the antioxidant system, including phenolic substances and antioxidant-related enzymes, to regulate postharvest fruit quality and stress responses (Marjorie et al., 2016; Serna-Escolano et al., 2019; Min et al., 2021). Studies have also shown that MeJA can affect the biosynthesis of fruit components, such as carotene, chlorophyll, and vitamins (Saniewski and Czapski, 1983; Pérez et al., 1993; Wolucka et al., 2005). In addition, Shi et al. (2021) showed that MeJA induced the accumulation of tea (*Camellia sinensis* L.) aroma precursors and improved the quality of fresh leaves. Moreover, it has been observed that the application of MeJA significantly enhanced the flavor quality of fruits and vegetables, such as peaches (*Prunus persica* L. Batsch), strawberries (*Fragaria* × *ananassa* Duch.), sweet basil (*Ocimum basilicum*), and tomatoes (*Solanum lycopersicum* L. cv. Messina) (Ziosi et al., 2008; Fernando et al., 2010; Zhang et al., 2012; Misra et al., 2014). Salicylic acid (SA) can maintain plant growth and development and respond to abiotic stress (Miura and Tada, 2014). Studies have reported that the application of MeJA and SA increased the amounts of bioactive substances in oil palm seedlings (*Elaeis guineensis* Jacq.) and mangosteen (*Garcinia mangostana* L.) during cold storage, thereby enhancing their antioxidant activity (Arthy et al., 2018; Mustafa et al., 2018a). The exogenous application of SA increased the stomatal conductance of barley (*Hordeum vulgare* L.) leaves under drought stress, thereby increasing dry matter content (Habibi, 2012). Huang et al. (2010) showed that SA treatment induces the accumulation of flavonoids and carotenoids, thereby enhancing the antioxidant activity of preharvest navel oranges (*Citrus sinensis* Osb.). Li et al. (2019) found that SA treatment increased the flavonoid content in tea. Martínez-Esplá et al. (2018) showed that preharvest SA treatment increases the antioxidant compounds of plums (*Prunus salicina* Lindl.).

Previous studies on MeJA and SA have mostly focused on stress tolerance, postharvest quality, and the preservation of fruits (Kim et al., 2009; Ibrahim et al., 2018; Se et al., 2020). Despite being a Chinese characteristic flavor vegetable, few studies have been conducted on the flavor quality and antioxidant activity of Chinese chive compared with those on that of tomatoes, onions, and garlic. As such, there are no data on the effects of exogenous hormones on the volatile components of Chinese chives, and there are few reports on the effects of the exogenous application of MeJA and SA on the growth physiology, quality, and antioxidant activity of Chinese chives. We hypothesized that the preharvest application of MeJA and SA promotes the growth of Chinese chive, improves the quality, volatile content, and antioxidant activity, thus improving the characteristic flavor, postharvest quality, preservation characteristics, and its commercial value. The objective of this study was to determine the effects of preharvest MeJA and SA treatments on the growth physiology, quality, volatile content, and antioxidant activity of Chinese chive and to identify suitable spraying concentrations of these compounds for this crop for better Chinese chive cultivation.

## Materials and Methods

### Plant Materials and Experimental Sites

The seeds of the Chinese chive cultivar “Chive God F1,” cultivated by the Fugou County Seed and the Seedling Research Institute of Henan Province, were used as the experimental material for this study. Seedlings were cultivated in Wushan (N 34°25′–34°57′, E 104°34′–105°08′), China, in the core demonstration area for Chinese chives, in May 2020. Wushan has a temperate continental semi-humid monsoon climate, with an annual average temperature of 10°C and a precipitation of 500 mm. The soil type at the test site was sandy loam, and the soil fertility was medium and uniform.

### Methods and Treatments

The experiment was set up in a completely randomized design with 11 treatments and three replicates. The plot size for each planting area was 2.7 m × 3.3 m (plant, 460 holes). On May 21, 2021, Chinese chive seedlings were transplanted into a plastic greenhouse (10 m × 30 m) with a row spacing of 20 cm, a hole spacing of 10 cm, and three seedlings per hole. Each plot was treated with exogenous hormones: an aqueous solution of MeJA (containing 0.1% ethanol and 0.1% Tween-20) at five concentrations: 50, 150, 300, 500, or 800 μM; an aqueous solution of SA (containing 0.1% ethanol and 0.1% Tween-20) at five concentrations: 50, 150, 300, 500, or 800 μM; and a control solution (0 μM; containing 0.1% ethanol and 0.1% Tween-20). Three liters of the aqueous solution of each treatment was sprayed on the leaves of the Chinese chive between 7:00 and 8:00 every morning. All treatments were applied for 3 days in a row (June 16, 2021 to June 18, 2021). After 35 days (June 25, 2021), the Chinese chive had attained the commercial standard, and plants from all plots were collected. Fifteen holes (three plants per hole) were randomly selected from each plot, with five holes for each of the three biological replicates. All samples were immediately frozen in liquid nitrogen and stored at −80°C prior to analysis.

### Growth and Photosynthetic Pigments Analysis

During harvest, 15 holes of Chinese chives (roots and shoots) were randomly selected from each plot within each treatment to record the fresh weight, and the dry weight was recorded after drying the plants at 105°C for 30 min, followed by 75°C for 72 h to a constant weight per a previously published method (Noreen et al., 2020).

The Chinese chive samples were taken using the same sampling method described above to determine the chlorophyll content. The photosynthetic pigments in the leaves were determined according to the method described by Arnon (1949) using the 80% acetone extraction technique. Briefly, a sample was obtained using a r puncher (0.5 cm diameter), 0.1 g of leaves was placed in a 25 ml test tube with a stopper, and 10 ml of 80% acetone was added, mixed well, sealed with a parafilm, and then chlorophyll was extracted in the dark for 48 h until the leaves turned white. The optical density of each extract was measured at 663 and 645 nm using a UV-1780 spectrophotometer [Shimadzu Instruments (Suzhou) Co., Ltd., Suzhou, China]. The Chl.a, Chl.b, and total Chls (Chl.T) were calculated with the following formulas:


$$\begin{array}{ccc} \text{Chl.a }\left({\text{mg g}}^{-1\;}\text{FW}\right) & = & [12.71\times \text{D663}-2.59\times \text{D645}]\times \left(\text{V/1000W}\right)\\ \text{Chl.b }\left({\text{mg g}}^{-1\;}\text{FW}\right) & = & [22.88\times \text{D645}-4.67\times \text{D643}]\times \left(\text{V/1000W}\right)\\ \text{Chl.T }\left({\text{mg g}}^{-1\;}\text{FW}\right) & = & [20.29\times \text{D645}+8.04\times \text{D663}]\times \left(\text{V/1000W}\right) \end{array}$$

Where V and W represent the total volume of extract (ml) and fresh weight of the sample (g), respectively.

### Nutritional Parameters Analysis

The vitamin C content was determined using the 2,6-dichloroindophenol stain method (Arya et al., 2000). The soluble protein content was determined using the Coomassie Brilliant Blue method (Bradford, 1976). The soluble sugar content was determined using the anthrone–sulfuric acid assay method (Wen et al., 2005). The nitrate content was determined according to Cataldo et al. (1975) with slight modifications.

### Total Phenol and Flavonoid Analysis

The total phenol content was determined using the Folin-Ciocalteu method with a slight modification (Hand et al., 2017). Briefly, a crude extract of phenols (0.2 ml) was obtained by extraction with 50% methanol (0.2 ml), which was mixed with distilled water (4.8 ml), followed by Folin-Ciocalteu’s phenol reagent (0.5 ml) and 20% sodium carbonate solution (2.5 ml). After incubating in water bath at 50°C for 30 min in the dark, the absorbance of the extract was measured at 760 nm using a UV-1780 spectrophotometer (Shimadzu Instruments, Suzhou, China). The results were compared with the calibration curve of gallic acid and expressed in milligrams of gallic acid equivalent (GAE) per gram of sample (mg GAE g^–1^).

The flavonoid content was determined following the method of sodium nitrite-aluminum nitrate (Chang et al., 2002) with a slight modification. A crude extract of phenol (1 ml) was obtained by extraction with 50% methanol, which was evenly mixed with 50% methanol (1 ml) and 5% ammonium nitrite (0.5 ml). After incubating for 5 min, 10% aluminum nitrate (0.5 ml) was added. After incubation for 6 min, 4% sodium hydroxide (2 ml) was added. After incubating for 10 min, the absorbance of the extract was measured at 510 nm using a UV-1780 spectrophotometer [Shimadzu Instruments (Suzhou) Co., Ltd., Suzhou, China]. The results were compared with the rutin calibration curve and expressed as rutin equivalent (RE) mg (mg RE g^–1^) per gram of sample.

### Electronic Nose Analysis of Volatile Content

A portable electronic nose (PEN3) electronic nose (Airsense Analytics GmbH, Schwerin, Germany) was used to analyze the volatile components of the samples. The PEN3 E-nose was equipped with a sensor array composed of 10 chemical sensing elements. The signal response of the sensor is expressed as (G/G0), which is defined as the ratio of the electrical conductivity of volatile matter to that of pure air (Cai et al., 2020). There were some differences in the types of sensitive substances, which could be detected by each sensor. Table 1 lists the substance types and performance descriptions of the 10 sensors. The electronic nose analysis of the volatile components of Chinese chive was performed according to Jia et al. (2020), with a slight modification. Chinese chives were fully ground (1.5 g) in a headspace bottle using 0.75 g anhydrous sodium sulfate and distilled water (2 ml). The bottle was then heated in a magnetic mixer at 70°C for 15 min to equilibrate the internal headspace gas, and then the injection needle was inserted into the headspace bottle for measurement of volatile compounds. The detection conditions were as follows: flush time of 60 s, sensor zero-time of 5 s, presample time of 5 s, injection flow rate of 400 ml min^–1^, and a measurement time of 120 s. Based on the data obtained at each time point, the average value of the stable maximum response of each sensor of three parallel samples was obtained, and a characteristic radar map was established.

**TABLE 1:** Substance type and performance description represented by 10 sensors of electronic nose.

Array serial number	Sensor name	Substance types	Sensor performance description
1	W1C	Aromatic	Aromatic components, benzenes
2	W5S	Broadrange	High sensitivity, sensitive to nitrogen oxides
3	W3C	Aromatic	Sensitive aromatic components, ammonia
4	W6S	Hydrogen	Mainly selective to hydride
5	W5C	Arom-aliph	Aromatic components of short-chain alkanes
6	W1S	Broad-methane	Sensitive to methyl groups
7	W1W	Sulfur-organic	Sensitive to sulfides
8	W2S	Broad-alcohol	Sensitive to alcohols, aldehydes and ketones
9	W2W	Sulph-chlor	Aromatic components, sensitive to organic sulfides
10	W3S	Methane-aliph	Sensitive to long-chain alkanes

### Enzymatically Produced Pyruvic Acid in Chinese Chive

The content of enzymatically produced pyruvic acid in Chinese chive leaves was determined according to the method of Liu et al. (2021) with a slight modification. Briefly, the sample was divided into two portions. One part was extracted by grinding with ultrapure water and quartz sand. Then, 1 ml of the extract was added to 2 ml 8% trichloroacetic acid and 1 ml 0.1% 2,4-dinitrophenylhydrazine and mixed well. The mixture was incubated in a water bath at 37°C for 10 min, and then 5 ml of 1.5 M NaOH was added. The other part of the sample was placed in a boiling water bath for 10 min to inactivate alliinase, followed by the same method as described for the other section to determine the background pyruvate levels. Absorbance was measured at 520 nm using a spectrophotometer. The enzymatically produced pyruvic acid was the pyruvic acid content in leaves minus the background pyruvic acid content. Pyruvate was determined using a standard sodium pyruvate curve.

### Antioxidant Parameters Analysis

The total antioxidant capacity of Chinese chive was determined according to the kit instructions (Suzhou Keming, Suzhou, China), including 2,2-diphenyl-1-picrylhydrazyl (DPPH) radical scavenging activity, ferric-reducing antioxidant power (FRAP), and 2,2’-azino-bis(3-ethylbenzothiazoline-6-sulfonic acid (ABTS) radical scavenging activity. Briefly, using a precooled mortar, the leaf tissue was ground (0.1 g) in 1 ml of precooled extract. The homogenate was centrifuged at 4°C at 10,000 × *g* for 10 min, and the supernatant was collected for testing. According to the instructions of the DPPH radical scavenging activity and FRAP kits, the respective solutions were mixed and allowed to react at room temperature in the dark for 20 min and at room temperature for 20 min. A microplate reader (SpectraMax i3, Molecular Devices, Sunnyvale, CA, United States) was used to measure absorbance at 515 and 593 nm. The same extraction method was used to extract the supernatant from the sample to determine ABTS radical scavenging activity. When the tested substance was added to the ABTS+ solution, antioxidants reacted with ABTS+ and faded the reaction system. The change in absorbance was measured at 734 nm. The antioxidant capacity of the Chinese chive was quantified by the change in absorbance and Trolox was used as the control system.

### Statistical Analysis

All experimental data were analyzed using IBM Statistical Product and Service Solutions (SPSS) Statistics version 21.0, and the statistical significance of treatment means was evaluated using Duncan’s multiple range test (*p* < 0.05). All data were presented as mean ± SE. Data figures, correlation analysis, and principal component analysis (PCA) were generated using Origin Pro (2019b).

## Results

### Effect of Methyl Jasmonate and Salicylic Acid Treatments on Dry and Fresh Weight of Chinese Chive

**FIGURE 1:**
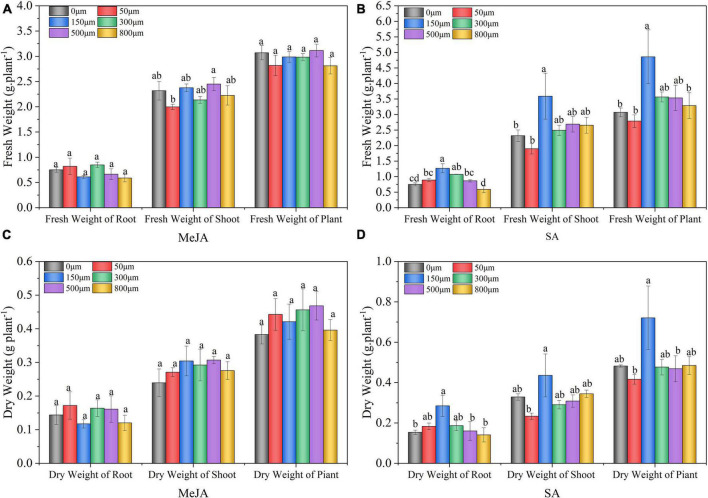
Fresh **(A,B)** and dry weight **(C,D)** of Chinese chives affected by methyl jasmonate (MeJA) and salicylic acid (SA) treatments. Data represent the mean ± SE (*n* = 3). Different lowercase letters indicate statistical significance by Duncan’s multiple range test (*p* < 0.05).

The application of SA treatment had a significant effect on the dry weight and fresh weight of the shoots and roots of Chinese chives (Figures 1B,D). The fresh weight of roots, shoots, and plants treated with 150 μM SA increased significantly by 69, 55, and 58%, respectively, and the dry weight increased by 85, 33, and 50%, respectively, compared with that of the control (0 μM). With the increase in the SA concentration, the dry and fresh weight of Chinese chives exhibited a decreasing trend. However, the application of MeJA had no significant effect on the dry and fresh weights of Chinese chives (Figures 1A,C).

### Effect of Methyl Jasmonate and Salicylic Acid Treatments on Photosynthetic Pigment Contents of Chinese Chive

**FIGURE 2:**
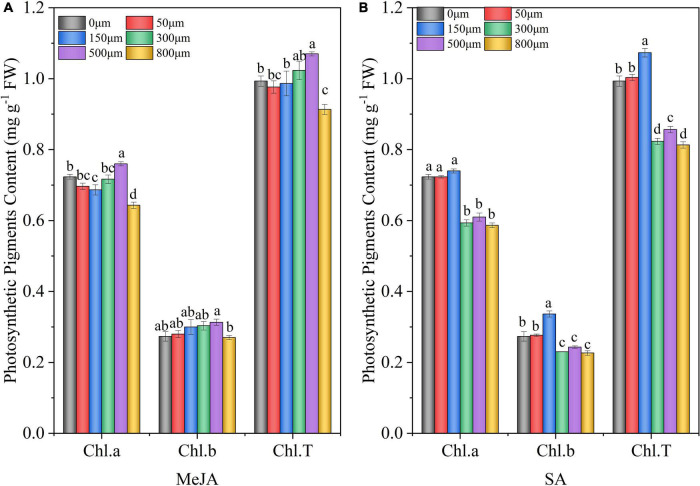
Chlorophyll (Chl) Chl.a, Chl.b, and Chl.T contents of Chinese chives, as affected by MeJA **(A)** and SA **(B)** treatments. Data represent the mean ± SE (*n* = 3). Different lowercase letters indicate statistical significance by Duncan’s multiple range test (*p* < 0.05).

Compared with the control (0 μM) treatment, 500 μM MeJA significantly increased Chl.a and Chl.T by 5 and 8%, respectively, in Chinese chives (Figure 2A); however, 800 μM MeJA significantly decreased Chl.a and Chl.T content. Treatment with 150 μM SA significantly increased Chl.b and Chl.T content by 23 and 8%, respectively, compared with the control (0 μM) (Figure 2B). When treated with 300–800 μM SA, the content of Chl.a, Chl.b, and Chl.T in Chinese chives decreased significantly compared with the control (0 μM).

### Effect of Methyl Jasmonate and Salicylic Acid Treatments on Vitamin C, Soluble Sugar, Soluble Protein, and Nitrate Content of Chinese Chive

**FIGURE 3:**
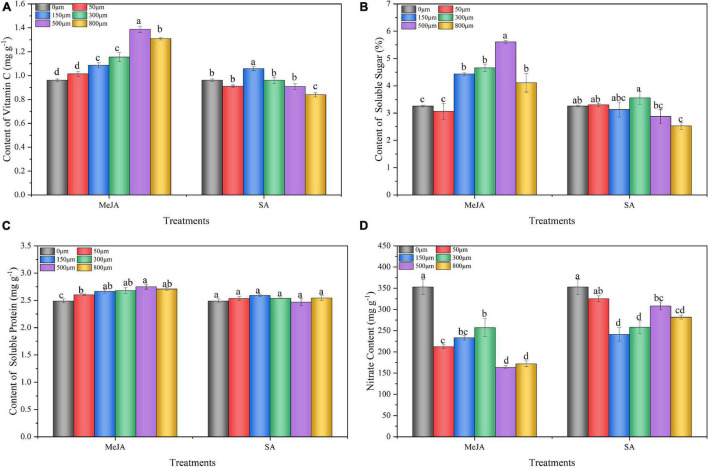
Vitamin C **(A)**, soluble sugar **(B)**, soluble protein **(C)**, and nitrate **(D)** contents of Chinese chives as affected by MeJA and SA treatments. Data represent the mean ± SE (*n* = 3). Different lowercase letters indicate statistical significance by Duncan’s multiple range test (*p* < 0.05).

The contents of vitamin C, soluble sugar, and soluble protein in Chinese chives treated with MeJA were significantly higher than those in the control (0 μM) (Figures 3A–C). The vitamin C, soluble sugars, and protein contents in Chinese chives treated with 500 μM MeJA significantly increased by 44, 10, and 72%, respectively, compared with those in the control (0 μM). However, 50–500 μM SA treatment had no significant effect on the soluble sugar content. SA at 800 μM significantly decreased the vitamin C and soluble sugar content of Chinese chives (Figures 3A,B); however, there was no significant effect on the soluble protein content (Figure 3C). Compared with the control (0 μM), treatment with SA (150–800 μM) and MeJA significantly reduced the nitrate content of Chinese chive (Figure 3D).

### Effect of Methyl Jasmonate and Salicylic Acid Treatments on Total Phenol and Flavonoid Content of Chinese Chive

**FIGURE 4:**
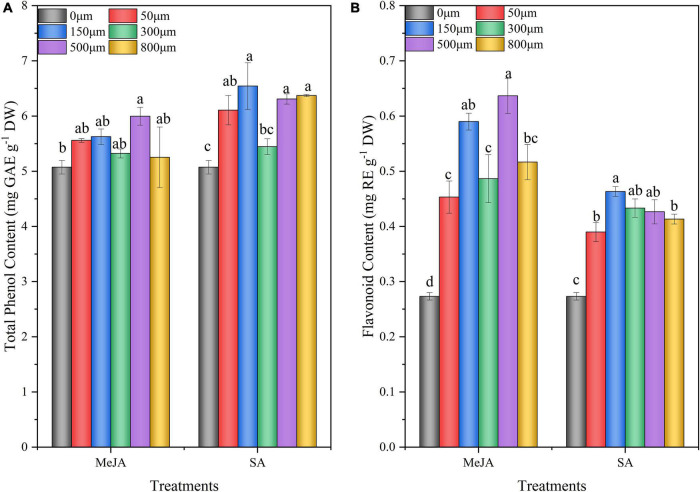
Total phenol **(A)** and flavonoid **(B)** content of Chinese chives as affected by MeJA and SA treatments. Data represent the mean ± SE (*n* = 3). Different lowercase letters indicate statistical significance by Duncan’s multiple range test (*p* < 0.05).

The methyl jasmonate and salicylic acid treatments had significant effects on the total phenol and flavonoid contents of Chinese chive (Figure 4). Treatment with different concentrations of SA significantly increased the total phenol content (Figure 4A). After 150 μM SA treatment, the total phenol content significantly increased by 29% compared with the control (0 μM). However, treatment with different concentrations of MeJA significantly increased the total phenol content of Chinese chive (18.2%) at a concentration of 500 μM. Treatment with different concentrations of MeJA and SA significantly increased the flavonoid content of Chinese chive compared with the control (0 μM) (Figure 4B). The flavonoid content in Chinese chive was significantly increased by 133% and 70 after treatment with 500 μM MeJA and 150 μM SA.

### Effect of Methyl Jasmonate and Salicylic Acid Treatments on Volatile Composition and Flavor Intensity of Chinese Chive

**FIGURE 5:**
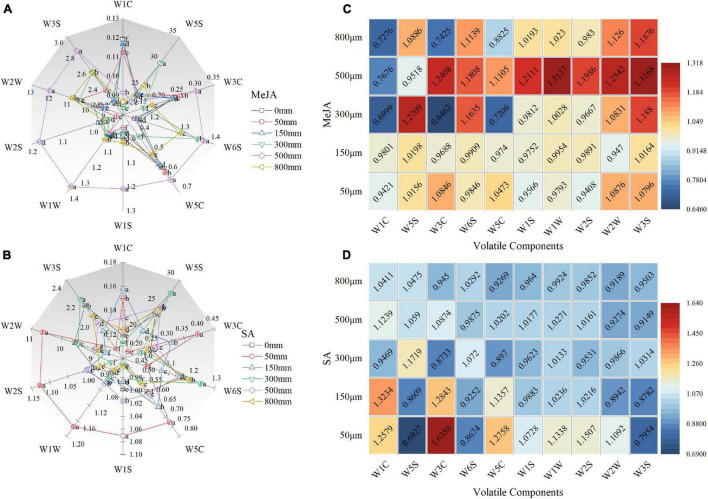
Radar map of volatile components of Chinese chives as affected by MeJA **(A)** and SA **(B)**. The radar map is represented by the signal response value (G/G0) of the electronic nose sensor, and G/G0 is defined as the ratio of the electrical conductivity of volatile matter to that of pure air. The data were normalized to 0 mm concentration. The increase or decrease in the amounts of volatile components after each treatment is compared with that in the control (0 μM). The color code shows that different numbers of volatile components were detected, and the proportion changes are related to the control (0 μM) level **(C,D)**. Data represent the mean ± SE (*n* = 3). Different lowercase letters indicate statistical significance by Duncan’s multiple range test (*p* < 0.05).

The 10 sensors of the electronic nose all responded to the volatile compounds of Chinese chive treated with different concentrations of MeJA and SA, and the responses of different sensors were differed (Figures 5A,B). The signal responses to the sensors were expressed by G/G0, which was defined as the ratio of the electrical conductivity of volatiles to that of pure air. The radar chart shows the response of the different sensors of the electronic nose to volatile substances in Chinese chive. In Figure 5, the relative resistivity (G/G0) values of the W5S and W2W sensors were higher than those of the other sensors, indicating that the volatile components of Chinese chive, such as nitrogen oxides, aromatics, and organic sulfides, were more sensitive to the sensor. As can be seen from the radar map, the contours of 500 μM MeJA and 50 μM SA were the largest. After treatment with different concentrations of MeJA and SA, the outline of the radar map gradually changed, indicating that the composition of volatile compounds in Chinese chives changed. In addition, the heat map showed an increase or decrease in volatile content after MeJA and SA treatments compared with the control (0 μM) treatment (Figures 5C,D). With the exception of W1C and W5S, the relative resistivity of the other eight sensors treated with 500 μM MeJA was increased compared with that of the control (0 μM) sensors, which indicated that treatment with 500 μM MeJA increased the amounts of most of the volatile components, such as sulfides, aromatics, and organic sulfides. Moreover, treatment with 50 and 150 μM SA increased the amounts of characteristic aromatic substances, such as aromatics and sulfides, compared with the control (0 μM).

The methyl jasmonate and salicylic acid treatments had a significant effect on the total flavor intensity (sulfur compounds) of Chinese chive, characterized by the pyruvate produced by alliinase (Table 2). Treatment with 500 μM MeJA and 50 and 150 μM SA significantly increased the enzymatic pyruvate content compared with the control (0 μM).

**TABLE 2:** Characteristic flavor of Chinese chives as affected by methyl jasmonate (MeJA) and salicylic acid (SA) treatments.

EPY (μg ml^–1^)	0 mm	50 mm	150 mm	300 mm	500 mm	800 mm
MeJA	16.5 ± 0.3d	29.9 ± 0.8a	23.3 ± 0.5b	24.3 ± 0.7b	32.0 ± 0.9a	20.5 ± 0.6c
SA	16.5 ± 0.3d	66.9 ± 0.4a	67.6 ± 0.5a	50.1 ± 1.7b	23.0 ± 0.5c	8.2 ± 0.3e

*Data represent the mean ± SE (n = 3). Different lowercase letters indicate statistical significance by Duncan’s multiple range test (p < 0.05).*

### Classification Model of Volatile Composition of Chinese Chive Using Principal Component Analysis

**FIGURE 6:**
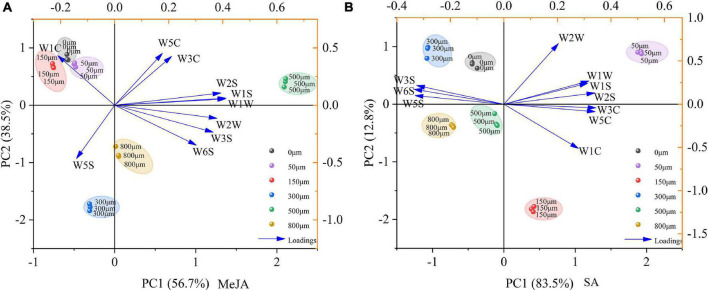
Principal component analysis (PCA) of volatile composition of Chinese chives after MeJA **(A)** and SA **(B)** treatments for a score scatterplot and a loading plot. PCA was based on the ratio of the electrical conductivity of volatiles to that of pure air (G/G0) in response to 10 sensors as variables. PCA aims to solve multicollinearity among multiple covariables, represents the differences between samples, and provides information from electronic nose pattern recognition files.

The principal component analysis was used to study the volatile components of Chinese chives treated with MeJA and SA (Figure 6). The first and second principal components captured most of the variation within the different MeJA and SA concentrations. The sum of the first two principal components treated by MeJA reached 95.2%, of which PC1 and PC2 explained 56.7 and 38.5% of the total variance, respectively (Figure 6A). The sum of the first two principal components treated by SA reached 96.3%, of which PC1 and PC2 explained 83.5 and 12.8% of the total variance, respectively (Figure 6B). In addition, it can be seen from the loading plot that W1W and W5C had strong loadings with the first and second principal component and W3C and W2W had strong loadings with the first and second principal component and thus were used as representative factors reflecting the volatile components of Chinese chives treated with different concentrations of MeJA and SA, indicating these treatments have considerable effects on the sulfide, organic sulfides, and aromatic volatile components of Chinese chive.

### Effect of Methyl Jasmonate and Salicylic Acid Treatments on the Antioxidant Activities of Chinese Chive

**FIGURE 7:**
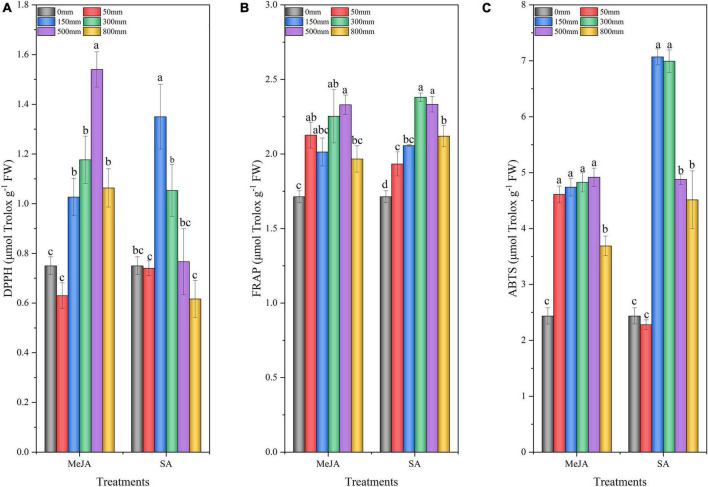
The 2,2-diphenyl-1-picrylhydrazyl (DPPH) radical scavenging capacity **(A)**, ferric-reducing antioxidant power (FRAP) **(B)**, and Trolox-equivalent antioxidant capacity (ABTS) **(C)** of Chinese chives as affected by MeJA and SA treatments. Data represent the mean ± SE (*n* = 3). Different lowercase letters indicate statistical significance by Duncan’s multiple range test (*p* < 0.05).

The methyl jasmonate and salicylic acid treatments had a significant effect on the antioxidant activity of Chinese chives (Figure 7). All antioxidant activity determination methods, such as DPPH radical scavenging activity (Figure 7A), FRAP (Figure 7B), and ABTS radical scavenging activity (Figure 7C), indicated significantly higher antioxidant activity in MeJA (150–500 μM) and SA (150 μM)-treated Chinese chives than in the control (0 μM). The highest scavenging capacity was recorded in Chinese chives treated with 500 μM MeJA and 150 μM SA. The scavenging ability of samples treated with 800 μM MeJA and 300–800 μM SA showed a downward trend.

### Correlation Analysis and Principal Component Analysis

**FIGURE 8:**
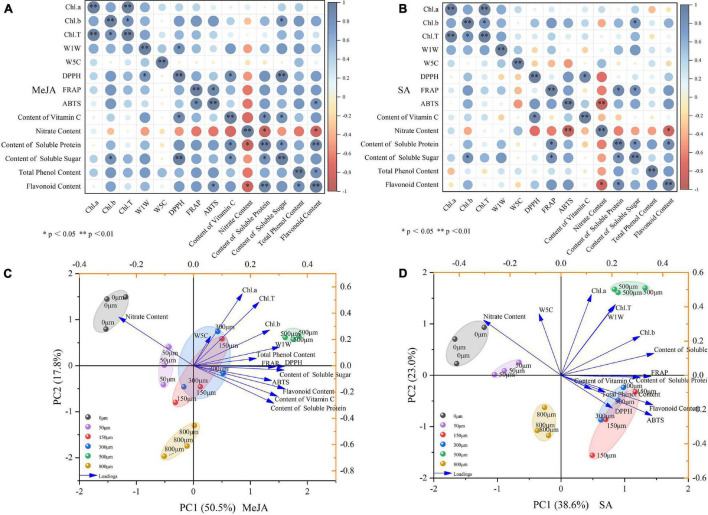
The heat map shows the correlation analysis between the observed parameters processed by MeJA **(A)** and SA **(B)**. The score plot and loading plot show the PCA analysis of the observation parameters processed by MeJA **(C)** and SA **(D)**. Pearson correlation analysis and PCA was carried out with chlorophyll, soluble protein, soluble sugar, total phenols, flavonoids, antioxidant activity, and volatile components as variables. ** and ** denote correlation coefficients that are significant at *p* < 0.05 and 0.01 level, respectively.

The correlation analysis of the parameters observed in this study showed that Chl.a had a significantly correlated with the Chl.T at *p* < 0.01 after MeJA and SA treatment, and Chl.b had a significantly correlated with soluble sugar and Chl.T at *p* < 0.5 (Figures 8A,B). DPPH had a significantly correlated with vitamin C and W1W after MeJA treatment (*p* < 0.5), and it had a significantly correlated with soluble sugar (*p* < 0.01); ABTS had a significantly correlated with FRAP and flavonoids (*p* < 0.5); soluble protein had a significantly correlated with flavonoids (*p* < 0.01); total phenols had a significantly correlated with flavonoids (*p* < 0.5) (Figure 8A); however, nitrate had a significantly negatively correlated with flavonoids and soluble proteins (*p* < 0.5). After SA treatment, the DPPH had a significantly correlated with vitamin C at *p* < 0.5; FRAP had a significantly correlated with soluble sugar and protein at *p* < 0.5; soluble protein had a significantly correlated with sugar and flavonoids at *p* < 0.5 (Figure 8B); however, nitrate had a negatively correlated with flavonoids (*p* < 0.5), and negatively correlated with ABTS (*p* < 0.01).

The PCA of the parameters observed in this study is shown in Figures 8C,D. The different concentrations of MeJA and SA treatments and various parameters constituted the corresponding groups. The sum of the first two principal components treated with MeJA reached 68.3% (Figure 8C). PC1 accounted for 50.5% of the total variance. PC2 accounted for 17.8% of the total variance. The sum of the first two principal components treated by SA was 61.3% (Figure 8D). PC1 accounted for 38.6% of the total variance. PC2 accounted for 23% of the total variance. In addition, it can be seen from the loading plot that flavonoids and Chl.a showed strong loadings with the first and second principal component, soluble sugar and Chl.a showed strong loadings with the first and second principal component and thus can be used as representative factors to reflect the effects of different concentrations of MeJA and SA on the growth physiology, quality, volatile content, and antioxidant potential of Chinese chive (Figures 8C,D).

## Discussion

Exogenous application of MeJA and SA can induce different biochemical, morphological, and physiological responses of plants (Huang et al., 2017). In our study, we found that MeJA had no significant effect on the fresh and dry weights of Chinese chives (Figures 1A,C). Our results contradict the findings of Baek et al. (2021), who reported that MeJA treatment increased biomass in tomatoes. However, Kurowska et al. (2020) showed that the application of MeJA significantly reduced the first leaf sheath and dry leaf weight of barley seedlings. This suggests that different plants, tissue types, and developmental stages respond differently to MeJA. SA promoted plant growth by improving plant photosynthesis and significantly promoted the fresh and dry weight of shoot and root (Gururani et al., 2013). Our results showed that SA (150 μM) treatment significantly increased the fresh and dry weights of root, shoot, and plants (Figures 1B,D). Since the treatments were applied during the peak growth period of Chinese chive, the increase in fresh and dry weight could be attributed to the effect of SA on alleviating biotic and abiotic stresses in the plant, thus increasing net photosynthetic rate and productivity (Hasanuzzaman et al., 2017). In addition, we observed that both 800 μM MeJA and SA inhibited the growth of Chinese chive. This may be due to the effects of high concentrations of MeJA and SA on plants similar to abiotic stress, reducing plant photosynthesis and thus inhibits plant growth (Kang et al., 2012; Kurowska et al., 2020).

Among studies on the effects of JA on plant photosynthesis, the most controversial research is the effect on photosynthetic plant pigments (Shu et al., 2016). *Exogenous JA significantly inhibited photosynthesis in broccoli* (Brassica oleracea L.) and *tomato*, but promoted *photosynthesis and chlorophyll accumulation in rapeseed (*Brassica napus L.*) (*Rahnamaie-Tajadod et al., 2017*;* Ding et al., 2018*)*. In the present study, we found that the total chlorophyll content after 500 μM MeJA treatment was significantly higher than that in the control (Figure 2A)*, which was similar to that* reported by Ji et al. (2018) *on maize seedlings (*Zea mays *L.). In addition, exogenous application of JAs increased photosynthetic pigments in a dose-dependent manner, thereby increasing photosynthetic efficiency (*Sirhindi et al., 2020*). The same results were shown in our study, where chlorophyll increased with increasing MeJA concentration (50–500* μ*M).* Previous studies have shown that *SA application increased the chlorophyll content of wheat (*Triticum aestivum *L*.*)* (Singh and Usha, 2003*). Our results showed that treatment with 150* μ*M SA significantly increased Chl.b and Chl.*T content (Figure 2B), *which was similar to that* reported by Zhang et al. (2021). *However, the high concentrations of MeJA (800* μ*M) and SA (300–800* μ*M) significantly reduced the total chlorophyll content. This may be due to the* fact that the *high concentrations of MeJA and SA can activate genes related to chlorophyll catabolism, which is related to the degradation of chlorophyll* (Zhu et al., 2015). *In addition, chlorophyll is the most prominent natural pigment in green vegetables and has significant antioxidant activity (*Lanfer-Marquez et al., 2005*). The chlorophyll can also reflect the senescence of plants and the maturity, color, quality, and freshness of fruits and vegetables through changes in its structure* (Wang et al., 2005).

The nutritional properties of vegetables are not only protective compounds that maintain their quality, but also guarantee nutritional value (Mustafa et al., 2018b). The preharvest treatment of exogenous MeJA and SA simulated biological stress and increased phytochemical compounds in different crops (Ku and Juvik, 2013; Sariñana-Aldaco et al., 2020). Our results showed that the contents of vitamin C, soluble sugar, soluble protein, total phenol, and flavonoid in Chinese chives treated with MeJA (500 μM) preharvest were significantly higher than those in the control (Figures 3A–C, 4), which was similar to the results of Min et al. (2021). The increase of total phenols and flavonoids in MeJA treatment may be due to the regulation of various physiological and metabolic processes, thus affecting the nutritional quality of crops. However, our results contradict the findings of Yamamoto et al. (2020), who reported that MeJA treatment significantly reduced flavonoids in citrus, indicating that MeJA has different regulation of bioactive substances in different crops, which is worthy of further study. SA affects the phenylpropanoid pathway by inducing its key enzymes phenylalanine ammonia lyase and chalcone synthase, resulting in the accumulation of phenolic compounds (Ghasemzadeh et al., 2012). In the present study, we found that SA (150 μM) treatment significantly increased the content of vitamin C, total phenols, and flavonoids in Chinese chive (Figures 3A, 4). Flavonoids are widely found in plants and play important roles in antioxidant, anticancer, antibacterial, and antimutagenic effects, and they are also one of the antioxidants produced by plants for their own survival (Treutter, 2006*;* Ghasemzadeh et al., 2010; Sharma et al., 2014). The MeJA and SA treatments increased the contents of total phenols and flavonoids in Chinese chives, thus improving the antioxidant activity of Chinese chives. In addition, we observed that both MeJA and SA treatments reduced the nitrate content of Chinese chives (Figure 3D). This may be because the reduction in nitrogen (N) uptake as an immediate consequence after MeJA treatment and the concomitant reactivation of endogenous N from leaves to roots, thereby reducing nitrate accumulation in leaves (Rossato et al., 2002). In general, preharvest MeJA (500 μM) and SA (150 μM) treatments improved the nutritional quality and safety quality of Chinese chives.

The MeJA treatment can trigger biosynthesis of volatile secondary metabolites and non-volatile secondary metabolites (Cheong and Choi, 2003). A sensor equipped with an electronic nose can simulate human olfactory factors and specific fingerprints of volatile tissues (Schaller et al., 1998; Benedetti et al., 2008). We detected volatile components of Chinese chives by electronic nose mainly nitrogen oxides (W5S), aromatics, and organic sulfides (W2W) (Figures 5A,B), which are more sensitive to the sensors of electronic nose and are characteristic aromatic substances of Chinese chives (Abbey et al., 2001). The volatile components of Chinese chives were dominated by methionine produced methyl sulfide, followed by Alliin produced allyl sulfide (Yabuki et al., 2010). The treatment with MeJA (500 μM) and SA (50 and 150 μM) increased the volatile composition of sulfur-containing compounds and enhanced the characteristic aromatic substances of Chinese chives. Meanwhile, we found that in addition to W1C and W5S, the relative resistivity of the other eight sensors treated with 500 μM MeJA was increased compared with that of the control sensors (Figure 5C), which indicated that treatment with this concentration of MeJA increased the content of most of the volatile components in Chinese chives, such as ammonia-based aromatic components (W3C), organic sulfides (W2W), hydrides (W6S), methyl groups (W1S), sulfides (W1W), alcohols, aldehydes, and ketones (W2S), short-chain alkane aromatic components (W5C), and long-chain alkanes (W3S). This may be because JA, as a lipid-based plant hormone, forms hydroperoxide through lipoxygenase pathway, and then oxidizes into short-chain aldehydes and ketones, increasing the hydride and aldehydes and ketones volatile components of Chinese chive (Li et al., 2010). Alcohols, aldehydes, and ketones have obvious odors (Tian et al., 2017), which may contribute greatly to the aroma of Chinese chive except aromatic hydrocarbons and sulfides. The aroma intensity of most long-chain alkanes is weak and contributes little to the aroma of food (Peng et al., 2014). However, compared with the control, 500 μM MeJA treatment increased the content of long-chain alkanes, which requires further study to determine the contribution of long-chain alkanes to aroma. The loading plots further indicated that different concentrations of MeJA and SA treatment had considerable effects on the volatile components compared with the control, such as sulfide, aromatic components (ammonia), short-chain alkane aromatic components, methyls, alcohols, aldehydes and ketones, organic sulfides (Figure 6). In addition, we found that 500 μM MeJA and 150 μM SA significantly increased the enzymatic pyruvate content (Table 2), which was consistent with the results of electronic nose testing that increased the content of organic sulfur compounds, thereby improving the flavor intensity of Chinese chives.

Previous studies have shown an increase in plant antioxidant activity after application of MeJA (Wang et al., 2008; Szymanowska et al., 2015), and this was similarly observed for DPPH, FRAP, and ABTS activity of the Chinese chive with MeJA application in this study (Figure 7). The improvement of antioxidant capacity of plants is related to the increase of phenols and other bioactive substances (Flores et al., 2015). In this study, the increase in the content of bioactive substances such as total phenols and flavonoids may be responsible for the improved scavenging and reducing abilities of Chinese chives after MeJA (500 μM) and SA (150 μM) treatments. However, this contradicts the study that Mustafa et al. (2016) reduced the content of antioxidants by applying MeJA in carambola (*Averrhoa carambola*). This result indicates that the JA signal is involved in the balance of the redox reaction (Dong et al., 2010), which is worthy of further study. The antioxidant activities (DPPH, ABTS, and FRAP) after MeJA treatment had a significant correlation with organic sulfur compounds, vitamin C, flavonoids, and soluble sugars (Figures 8A,B). SA processing showed similar results. This further indicated that the improvement of antioxidant capacity of Chinese chive could be closely related to the increase in the content of bioactive substances. JA, MeJA, and SA are the most widely used elicitors that promote the production of more bioactive compounds in plants, which in turn improve the nutritional quality and antioxidant activity of plants (Baenas et al., 2014). PCA analysis showed that MeJA (500 μM) and SA (150 μM) positively regulate Chl.a, Chl.T, Chl.b, sulfides, total phenols, FRAP, DPPH, soluble sugar, ABTS, flavonoids, vitamin C, and soluble protein. In general, MeJA and SA enhance the bioactive substances content and antioxidant activity, thereby improving the medicinal value, postharvest quality, and preservation characteristics, thus increasing the commercial value of Chinese chives.

## Conclusion

Our results suggest that preharvest treatments with 500 μM MeJA and 150 μM SA significantly increased the content of total chlorophyll, soluble sugar, total phenols and flavonoids, volatile components, and antioxidant activity of Chinese chive. In addition, 500 μM MeJA and 150 μM SA significantly increased the soluble protein content, and the dry and fresh weight, respectively. Simultaneously, 500 μM MeJA and 150 μM SA significantly increased the enzymatic pyruvate content, effectively increased the amounts of sulfide and aromatic volatile components, and improved the characteristic flavor compounds of Chinese chive. In addition, the antioxidant activity was improved after preharvest treatment with 500 μM MeJA and 150 μM SA, which could improve the postharvest quality and preservation characteristics, and therefore enhance the commercial value. In conclusion, preharvest treatment with 500 μM MeJA and 150 μM SA could be the best option for improving the growth physiology, quality, antioxidant activity, and the flavor of Chinese chive.

## Data Availability Statement

The original contributions presented in the study are included in the article/supplementary material, further inquiries can be directed to the corresponding author/s.

## Author Contributions

CW, JZ, JX, and JY conceived and designed the experiments. CW, JZ, and YG analyzed the data. CW wrote the manuscript. CW, JZ, JLi, TN, and JLv were involved in the related discussion. JX, JZ, and BP helped to improve the quality of the manuscript. All authors have read and agreed to the published version of the manuscript.

## Conflict of Interest

The authors declare that the research was conducted in the absence of any commercial or financial relationships that could be construed as a potential conflict of interest.

## Publisher’s Note

All claims expressed in this article are solely those of the authors and do not necessarily represent those of their affiliated organizations, or those of the publisher, the editors and the reviewers. Any product that may be evaluated in this article, or claim that may be made by its manufacturer, is not guaranteed or endorsed by the publisher.
